# Listen to the shopkeeper

**DOI:** 10.7554/eLife.87366

**Published:** 2023-03-16

**Authors:** Rafael Maciel-de-Freitas

**Affiliations:** 1 Laboratório de Transmissores de Hematozoários, Instituto Oswaldo Cruz, Fiocruz Rio de Janeiro Brazil; 2 Department of Arbovirology, Bernhard-Nocht Institute for Tropical Medicine Hamburg Germany

**Keywords:** sparks of change, research culture, brazil, fieldwork, mosquitoes, community engagement, vector control

## Abstract

His mosquito control project heading for failure, a field entomologist recalls how a chance encounter led to a Eureka moment.

My country, Brazil, has the highest prevalence of dengue fever in the world, with over a million cases last year alone. In most cities it may be impossible to find a resident who has never been infected by a mosquito-borne virus. We still don’t have antiviral drugs or vaccines for many of these diseases, and the best approach is often to target the mosquitoes which spread them. I have devoted my career to understanding and controlling how these insects transmit illnesses. One of the first things I learnt is that this work can’t succeed without involving local communities.

Traditional methods of mosquito control often have limited success. Brazilian *Aedes aegypti* mosquitoes, which transmit many of these deadly viruses, have now become resistant to most insecticides. Meanwhile, drug dealers or uncooperative residents make it difficult for health inspectors to access and eliminate breeding sites in certain areas. I was therefore excited when, in 2012, I joined a team of 25 international scientists to pilot an approach never been tried before in Latin America: we were going to release lab-raised mosquitoes which carried the bacteria *Wolbachia pipientis*. In other parts of the world, this strategy had led to the incidence of dengue and chikungunya dropping by 70%.

**Figure 1:**
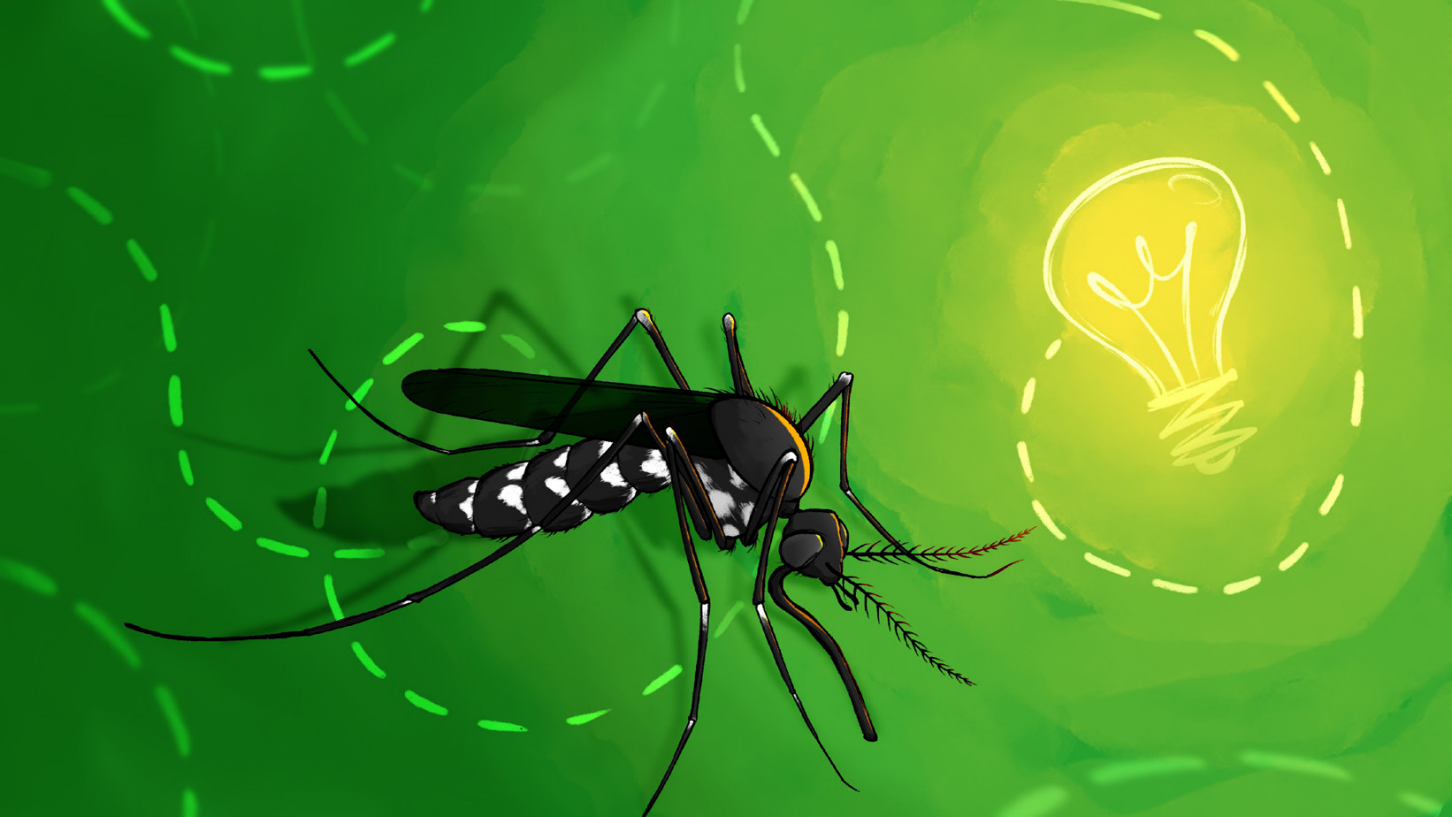
Controlling mosquito populations is a public health priority in Brazil.

Image credit: Marzia Munafo; @munafomarzia (CC BY 4.0)

*Wolbachia* colonises the cells of many insect species, but its presence in mosquitoes is associated with a decreased risk of transmitting diseases to humans. The bacteria can be introduced into a native population by releasing infected mosquitoes bred in the lab. As they mate with their wild counterparts, the females pass on the infection to the next generation: over time, the prevalence of *Wolbachia* increases and becomes stable, rendering the entire population less likely to infect people. For me and my colleagues, this initiative was a ray of hope. I was going to be the leader of the field entomology team, and I couldn’t wait to get started.

Our first task was to choose where the release would take place. We decided on Tubiacanga, an isolated neighbourhood of about 3,000 people on the northern coast of the city of Rio de Janeiro. Home to a small community of fishermen, its simple houses and unpaved streets reminded me of my childhood. Before we could go forward, however, we had to ensure that the residents were on board. They needed to be involved from the start so we could define priorities and plan the releases together. My colleagues in the community engagement team got to work, organising lectures at schools or health units, and going door-to-door to explain and discuss the project. Seven residents stepped forward to form a community reference group, and we went to meet with them every month to share updates and talk through concerns. In the end, independent surveys revealed that 88% of local householders supported the *Wolbachia* releases.

After more than two years of work, in September 2014 we were finally ready to go. Every morning for the next 20 weeks, our team would walk through the streets of Tubiacanga, stop in front of every four houses, and release 50 *Wolbachia*-carrying mosquitoes. In parallel, we immediately started to monitor the spread of the bacteria in the population. For the first six weeks things seemed to be on track, but this all changed around week seven. We knew from data gathered in Australia, Vietnam and Indonesia that the infection rate should have soon peaked at around 80%, but instead, week after week, it plateaued at 40%. We couldn’t understand why this was happening.

Trying to solve this problem brought some sleepless nights for me and my team. One by one, we considered and tested several hypotheses. We checked that our lab-raised insects were big enough to survive in the wild (they were), and that we weren’t slowing the spread of *Wolbachia* by releasing more males than females (we weren’t). We examined whether the bacterium was somehow lost in the released insects (it wasn’t). We trialled releasing twice the number of insects every week, to no avail. We were back to square one.

We weren’t the only people to be frustrated by the lack of results. Residents had always been highly supportive but having 15,000 new mosquitoes appear in front of their houses every week was bound to annoy them. Our insects were less likely to transmit disease yet the females still bit, fed on blood and bothered people. We were working closely with the community engagement team to be transparent and explain what was happening. We started to avoid the houses of those who had complained, and we moved the releases to five in the morning, when people would be sleeping with their doors and windows closed. Yet despite their trust, it was becoming clear that the residents were unsatisfied and frustrated, especially those with elderly relatives or babies at home.

It all came to a head during the last weeks of the release period. After each 5am release, the entomology team used to have a coffee break at the local grocery store before going back to the lab. It was not unusual for people to approach us there to complain about the project, which we couldn’t do much about since we had not figured out yet what was happening. One morning, the owner of the store came to us to ask whether we belonged to the mosquito project. Stressed and exhausted, we just nodded in silence and braced ourselves for more complaints. Instead, he smiled and begged us to never stop the releases. *That* response was definitively unexpected. When I asked why he felt this way, he enthusiastically replied: “I will become rich! People are buying more insecticides than ever.”

And here it was, our Eureka moment. We had assumed that our mosquitoes were resistant to insecticides, having been bred from a Brazilian stock. What if something had gone wrong? Unlike Archimedes, we didn’t run naked through the streets of Tubiacanga – instead, we immediately went back to the lab.

Soon, genetic analyses confirmed our suspicion. Our *Wolbachia*-infected colony had been created using Brazilian mosquitoes which could survive insecticides, but the population had been maintained for 17 generations while we waited for the release to be approved. Insecticide resistance is costly to the organism, and while it is useful in the wild, there is no need for it in a lab environment. Despite regularly adding wild males to the colony, the trait had been quickly selected against. In the end, we had released mosquitoes susceptible to insecticides in a native population that was highly resistant. Householders were buying sprays en masse to protect themselves, selectively killing our precious *Wolbachia*-carrying insects in the process.

Now that we knew the root of the problem, we decided to try again. We changed our breeding protocol to correct the issue and we went back to the community to explain why the project hadn’t worked in the first place; they decided to give us a second chance. Eight months after the end of our first failed attempt, we released our new *Wolbachia*-carrying mosquitoes in the neighbourhood and started the monitoring. We knew another failure would permanently bury the initiative. A few weeks in, the results sent shivers down my neck; we had finally reached an infection rate of 80%. The project had worked! In fact, seven years after the last release, *Wolbachia* is still present in over 95% of local mosquitoes. The lessons we learnt in Tubiacanga have since been applied in cities across Brazil – and the members of the community reference group still invite us for coffee when we are back in the neighbourhood.

The past few years have seen science denialism rising across the globe. In Brazil, there has been widespread debates about the safety of COVID vaccines, the Earth being round or whether satellites images of the Amazon burning are real. We need to rethink our way to communicate research beyond the lab and engage with citizens. Our release project taught me that, given the chance, everybody can contribute to science. The grocery store owner was by nature a good observer; I had seen him at several community meetings, and he was very proud of the fact that the first *Wolbachia* release in Latin America would take place in his neighbourhood. Without his input, *Wolbachia* would have never been a success story in his community.

## Share your experiences

This article is a Sparks of Change column, where people around the world share moments that illustrate how research culture is or should be changing. Have an interesting story to tell? See what we’re looking for and the best ways to get in touch here.

